# Assessing the Impact of Diet on the Mucosa-Adhered Microbiome in Piglets Using Comparative Analysis of Rectal Swabs and Colon Content

**DOI:** 10.3389/fmicb.2022.804986

**Published:** 2022-02-22

**Authors:** Raka Choudhury, Michiel Kleerebezem

**Affiliations:** Host-Microbe Interactomics Group, Department of Animal Sciences, Wageningen University & Research, Wageningen, Netherlands

**Keywords:** rectal swab, colon, mucosity factor, mucosa-adhered microbiome, gut microbiota, pig, solid feed

## Abstract

Previously, we demonstrated that rectal swabs provide a legitimate alternative to faecal sampling for the assessment of the intestinal microbiota in young piglets. However, we also reported that mucosa-adhered microbial populations were more represented in rectal swabs compared to faecal samples, albeit to a degree that varied per swab-sample. Here, we explored the possibility to exploit this variable enrichment of adhered populations in the rectal swabs to assess the impact of diet on mucosa-adhered microbiota in pre-weaning piglets. Paired samples of rectal swabs and colon luminal contents were collected from piglets just before weaning during two independent but similarly designed animal experiments [*n* = 28 piglets (experiment 1); *n* = 16 piglets (experiment 2)], with an early feeding treatment (EF) group that had access to customised fibrous feed in addition to sow’s milk and a control (CON) group exclusively reared on sow’s milk. The intestinal microbiome composition in rectal swabs and colon samples collected at 29 days of age were subjected to metataxonomic analysis. The results identified the genera *Escherichia-Shigella, Anaerococcus, Peptostreptococcus, Enterococcus, Trueperella*, *Actinomyces*, and *Peptoniphilus* as discriminative taxa enriched in rectal swabs compared to colon. Apart from *Escherichia-Shigella* (10–11% average relative abundance), most of these mucosa-adhered microbial genera display relatively low abundance. Rectal swab microbiota was found to be more variable, which is likely due to variable enrichment of mucosa-adhered microbes. Although almost exclusively driven by one of the experiments, the post-weaning diarrhoea-associated taxa *Escherichia-Shigella*, was enriched in CON compared to the EF group, suggesting that early life feeding may suppress post-weaning-diarrhoea-related problems in piglets. Our findings demonstrate that rectal swabs allow the investigation of the mucosa-adhered microbial populations as a function of dietary treatment in piglets. This offers opportunities to further study dietary approaches that suppress the abundance of the post-weaning diarrhoea associated adherent microbes like *Escherichia-Shigella*. Furthermore, we demonstrate that the paired swab-colon microbiota information (obtained from a subset of animals) can predict the mucosa-adhered populations or “mucosity factor” in rectal swab samples, facilitating the analysis of the adhered microbiota in large animal cohort studies using readily obtainable rectal swabs.

## Introduction

Choice of a suitable sample type is important for reliable microbiota analysis. This is because microbiome composition can be influenced by the chosen sample type, due to high niche-specificity of residing microbial communities. For instance, multiple niches or micro-environments exist in the intestinal tract, such as the intestinal lumen, epithelial mucus layer, or the mucosal tissues, which encompass differential abundance of microbial populations (Jones et al., 2018). This notion is supported by several studies that reported differences in the microbial populations found to be mucosa-associated or in the lumen of the gut in various mammals, including humans (Carroll et al., 2010; Burrough et al., 2017; Jones et al., 2018; Zhang et al., 2018; Mottawea et al., 2019; Wu et al., 2020; Klymiuk et al., 2021). These microbiota differences in different intestinal niches is likely to be dependent on variations in the niche-specific physical-chemical conditions, like substrate availability, oxygen concentration, pH, and is also influenced by interactions between the residing microbes (Yasuda et al., 2015; Pereira and Berry, 2017).

Faecal samples are commonly used to study gut microbiota as they are relatively easy to obtain. However, collecting faecal samples might not be feasible in all circumstances, especially involving neonatal piglets (Choudhury et al., 2019). Furthermore, faecal microbiota changes might not represent what is happening at the mucosal surface where bacteria interact more intimately with the host (Hollister et al., 2014; Burrough et al., 2017). Nevertheless, we previously confirmed the legitimacy of rectal swabs as an alternative sampling method to study the porcine microbiome development in early life, when faecal sampling is challenging and has a low success-rate. In the same study, we also implied that contrary to faecal samples, rectal swabs have the potential to also reflect the mucosa-adhered population albeit to an unpredictable and quite variable degree per sample (Choudhury et al., 2019).

In the present study, we aim to exploit this proposition by specifically investigating the mucosa-adhered populations in rectal swabs by comparative microbiota analysis with paired colonic samples (collected at the same time-point). This allowed us to evaluate the impact of early life feeding (fibrous-feed supplementation) on the mucosa-adhered microbiota in piglets, particularly taxa such as *Escherichia-Shigella* which could be relevant especially in the pig industry for tackling post-weaning stress and diarrhoea. Importantly, we demonstrate that the mucosa-adhered microbiota population can be predicted in a swab sample employing swab-colon distance information from a subset of animals.

## Materials and Methods

### Animals, Experimental Design and Sampling

The Animal Care and Use Committee of Wageningen University & Research (Wageningen, Netherlands) approved the protocol of the experiment (AVD104002016515), and is in accordance with the Dutch law on animal experimentation, which complies with the European Directive 2010/63/EU on the protection of animals used for scientific purposes. As described in our previous study (Choudhury et al., 2021b), the experiment was conducted with 12 multiparous Topigs-20 sows (range parity: 3–5) and their new-born piglets, co-housed (until weaning) at research facility Carus (Wageningen University & Research, Netherlands). The litters were divided into two experimental groups, early fed group (EF; *n* = 6 litters) and control group (CON; *n* = 6 litters). From 2 days onward, piglets belonging to the EF group were given the opportunity to forage on customised fibrous feed *ad libitum* in addition to suckling sow’s milk whereas the CON group nursed on sow’s milk only. Additional details of the diet, housing and management have been previously described (Middelkoop et al., 2020). Eating behaviour of individual piglets was assessed as previously described (Choudhury et al., 2021a). Briefly, from daily video recordings the amount of time spent eating or “eating time” was evaluated. Since the eating behaviour observational measurements may have some degree of subjectivity, they were considered as an “estimate” for the amount of eating per piglet. However, as reported earlier, we have observed eating behavioural scores and the feed-intake at litter level to be strongly correlated, supporting the legitimacy of the eating behaviour as an indicative quantification of eating, in addition of eating behaviour being strongly associated with (diet-induced) microbiome changes (Choudhury et al., 2021a,b).

To assess mucosa-adhered and luminal microbiota composition, paired colon (representing luminal) and rectal swabs (representing both luminal and mucosa-adhered) were collected from individual piglets (*n* = 28; 14 piglets per treatment) just before weaning (day 29). The choice of colon sample was justified by prior studies that showed that the colonic microbiota composition in pigs is quite similar in proximal and distal locations of the intestinal tract (Looft et al., 2014; Zhao et al., 2015; Crespo-Piazuelo et al., 2018; Gresse et al., 2019). The selection of sacrificed (subset) piglets were made by the following criteria: (a) no antibiotic treatment (b) close to mean body weight of the litter (c) close to average weight of the treatment group (d) one to three piglets per litter (e) equal male to female ratio. Rectal swab samples were obtained from piglets by inserting a sterile cotton swab (Puritan Medical, Guilford, ME, United States; Cat Number-25-3306-U) 20–30 mm into the rectum and rotating the swab against the bowel wall before placing it into a 5 ml Eppendorf tube. The samples were kept on ice during transport to the laboratory and stored at −20°C until further processing. Piglets were euthanised by intravenous injection of 20% sodium pentobarbital (EUTHASOL, 500 mg/mL, AST Farma B.V., Oudewater, Netherlands). Colon luminal contents were collected from the mid-colon intestinal segment, immediately frozen in liquid nitrogen and stored at −80°C until further processing. Rectal swab and colon samples collected from another (replicate) experiment just before weaning (29 days of age; *n* = 16, 8 piglets per treatment) with similar study design, were employed to reassess our findings in the first experiment. The early feeding diet were identical in the two experiments, although it started between 2 and 5 days of age after birth.

### DNA Extraction and 16S Sequencing

DNA extraction was performed by the repeated bead beating method (Yu and Morrison, 2004) using QIAamp DNA Stool Mini Kit (Qiagen, Hilden, Germany) according to the manufacturer’s instructions. For rectal swabs, 500 μl of lysis buffer (from the kit) was added to the 5 ml Eppendorf tube (holding swab) to obtain swab solution, which was used as a starting material for DNA extraction. Approximately 300 mg of luminal colon content (wet weight) was used for microbial DNA extraction. Both sample types were treated exactly the same during processing. The quality and quantity of extracted DNA samples were checked by gel electrophoresis (only representative samples) and NanoDrop DeNovix DS-11 Spectrophotometer (DeNovix Inc., Wilmington, DE, United States), respectively.

As described previously (Choudhury et al., 2021a), V3–V4 region of the bacterial 16S rRNA gene was PCR amplified (Bio-Rad C1000 thermal cycler, Bio-Rad Laboratories, Veenendaal, Netherlands) using V3F primer (5′-CCTACGGGNGGCWGCAG-3′) and V4R primer (5′-GACTACHVGGGTATCTAATCC-3′), 5′-extended with extension-PCR-adapters 5′-TCGTCGGCAGCGTCAGATGTG TATAAGAGACAG-3′ and 5′-GTCTCGTGGGCTCGGAGAT GTGTATAAGAGACAG-3′, respectively. Amplicons were purified using MSB Spin PCRapace (STRATEC Molecular, Berlin, Germany) and were sequenced at BaseClear B.V. (Leiden, Netherlands) using Illumina MiSeq system (paired end reads; 2 bp × 300 bp). Subsequently, a BaseClear in-house filtering protocol was applied for removal of reads containing adapters (up to minimum read length of 50 bp) and/or PhiX control signal, to generate the FASTAQ data file used for microbiota analysis. For the rectal swab/colon samples collected in replicate experiment 2, library construction of the V3–V4 hypervariable region [using primer set 341F/806R (341F: 5′-CCTAYGGGRBGCASCAG-3′, 806R: 5′-GGACTACNNGGGTATCTAAT-3′)] followed by sequencing on an Illumina HiSeq 2500 platform (paired end reads; 2 bp × 250 bp) were performed at Novogene (Novogene Co., Ltd., China). Negative controls were included during sample processing and sequencing steps, however, these samples did not generate 16S amplicons and did not pass sequencing quality control steps or generate data.

### Metataxonomic Analysis

Illumina reads were imported into the CLC Genomics Workbench version 11.01 and were processed using the CLC Microbial Genomics Module version 2.5.1 (CLC bio, Aarhus, Denmark). The paired end reads were merged into one high quality representative by CLC Workbench (Mismatch cost = 1, Minimum score = 40, Gap Cost = 4, Maximum unaligned end mismatches = 5). The CLC pipeline was used for primer and quality trimming. The remaining high-quality sequences were clustered into operational taxonomic unit (OTUs) at 97% identity threshold using SILVA database v132 (released on December 13, 2017) (Quast et al., 2013).

Multivariate principal component analysis (PCA; unsupervised), redundancy analysis (RDA; supervised), and partial redundancy analysis (pRDA; supervised and covariate corrected) were employed to identify microbiota signatures in sample types using CANOCO 5 (Microcomputer Power, Ithaca, NY, United States) (ter Braak and Smilauer, 2012). Furthermore, principal coordinate analysis (PCoA; squared Bray Curtis distance) was performed to assess the variation between the paired sample types collected from the same animals. The relative abundance of swab-associated (or mucosa-adhered) microbial groups (identified in RDA; top 20 microbes) were visualised in a heat map using Perseus 1.6.1.1 (Euclidean distance; average linkage method) (Tyanova et al., 2016). Comparison of the bacterial taxa relative abundance were performed by Mann–Whitney *U*-test using GraphPad Software 9 (San Diego, CA, United States^1^). The level of statistical significance was set at *P* < 0.05, whereas a trend was defined as 0.1 > *P* ≥ *0.05.* For assessing relationships between variables, Pearson/Spearman correlations were calculated in GraphPad Software 9 depending on the normal distribution of the data (passed Shapiro–Wilk test). To evaluate the impact of pre-weaning dietary intervention on the mucosa-adhered population in rectal swabs, pRDA was performed corrected for the sample type variation (squared Bray Curtis distance to its paired colon sample) and the “study” variable, when both the experiments were combined.

Our analyses illustrate that the presence of mucosa-adhered population in an individual swab sample is reflected by its “Bray Curtis distance” to the paired colon sample, which we refer to as the “mucosity factor” of the swab sample. We employed RDA analysis to create a swab-microbiota based ordination space that uses the “Bray Curtis distance to its paired colon microbiota” (i.e., the mucosity factor) as an explanatory variable. The ordination space created can subsequently be used to place additional swab samples that lack a paired colon counterpart as supplementary samples, enabling the prediction of their mucosity factor by extracting their CaseR scores in the ordination space created (which we refer to as “predicted mucosity factor”). The supplementary samples do not influence the created ordination space. CaseR scores of individual samples are the sample positions in the mucosity-ordination plot, derived from microbial response variables during redundancy analysis.

## Results

### Rectal Swab vs. Colon Microbiome Composition

Previously we have shown that rectal swab is a legitimate alternative to faeces, to study the early life microbiome development in piglets, although we also recognised that the mucosa-adhered microbial groups were more represented in rectal swabs to a variable degree (Choudhury et al., 2019). The present study aims to bring our previous findings one step further, by evaluating the impact of pre-weaning fibrous diet (Choudhury et al., 2021a) on the mucosa-adhered microbial population using paired colon and rectal swab samples obtained from the same piglets at the end of lactation.

**FIGURE 1:**
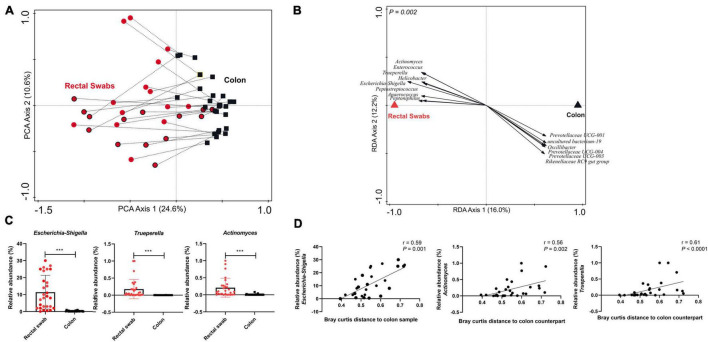
Evaluating microbiota composition (genus level) in paired rectal swabs and colon samples, collected from the same animals at weaning (29 days of age). **(A)** Principal Component Analysis (PCA) of rectal swab (red circles; early fed piglets: red circles with black border) and colon (black squares) microbiota, with paired samples (i.e., collected from the same animal) joined by dotted lines. **(B)** Redundancy analysis (RDA) of rectal swab and colon samples (adjusted explained variation = 14.5%; *P* = 0.002), displaying discriminating microbial groups (response score >0.60). **(C)** Bar plots displaying relative abundance of representative microbes enriched in the rectal swab samples compared to the colon samples (Mann–Whitney *t* test; ***P < 0.0001). **(D)** Spearman correlation between the relative abundance of representative microbes in the rectal swab sample and the (squared Bray Curtis) distance of the rectal swab with respect to its paired colon sample.

Global comparison by principal component analysis revealed that samples predominantly cluster on basis of sample-type, although a few paired samples (swab and colon samples obtained from the same animal) appeared particularly similar to each other (Figure 1A). Higher variability was observed within the swab samples (reflected by the intra-sample distance in the plot; Supplementary Figure 1A) compared to the colon samples. Colon seemed to have a higher alpha diversity compared to rectal swabs (Supplementary Figure 1B). Both sample types are characterised by distinct microbial signatures that were identified using RDA (Figure 1B and Supplementary Figure 2). Swab enriched genera included *Escherichia-Shigella, Enterococcus, Actinomyces, Trueperella, Peptostreptococcus, Anaerococcus, Peptoniphilus*, whereas *Oscillibacter, Prevotellaceae UCG-004, Prevotellaceae UCG-003 and Rikenellaceae RC9 gut group* were more abundant in the colon sample. Most of these swab-associated microbial groups were previously reported to represent mucosa-adhered microbes that inhabit mucosal surfaces in the gastrointestinal tract (Murphy and Frick, 2013; Mann et al., 2014; Könönen and Wade, 2015; Kelly et al., 2017; Rzewuska et al., 2019). Looking at the nature of the swab samples, it makes sense that the contrast with the luminal microbiota is reflecting the mucosa-adhered microbiota that are collected by rectal swabs. Notably, apart from *Escherichia-Shigella* (ranging from 0.07 to 30%), most of these mucosa-adhered microbial genera display relatively low abundance (Figure 1C and Supplementary Figure 1C). Importantly, there were significant correlations between the relative abundance of the swab-enriched genera and the Bray Curtis distance of the swab microbiota and its paired colon microbiota (Figure 1D and Supplementary Figure 1D). Thereby this distance metric appears to reflect the sample-specific and variable representation of mucosa-adhered microbes in the microbiota collected by rectal swabs.

To confirm our findings, we analysed a set of paired swab-colon samples collected in a second experiment having a similar study design (experiment 2; independent biological replicate experiment), reaching very similar conclusions about the variable degree of representation of the mucosa-adhered microbial groups in rectal swabs, and corroborating the finding that the *Escherichia-Shigella* genus is a dominant member of the piglets’ adherent microbiome (Supplementary Figures 3A–D). Although the two experiments differed in their microbiota composition (not shown), we observed similarity with regard to the significant correlation (*r* = 0.35; *P* < 0.0001) between the microbial response scores obtained from the RDA that demonstrated distinct microbial signatures in rectal swabs and colon (Supplementary Figure 3E).

### Assessing Mucosa-Adhered Population in Rectal Swab of Early Fed Piglets

**FIGURE 2:**
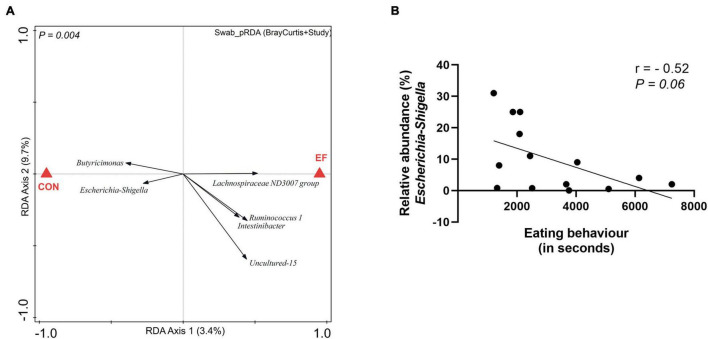
Impact of nutritional intervention on mucosa-adhered population. **(A)** Partial redundancy analysis (pRDA) of the dietary intervention (EF vs. CON) employing the rectal swab microbiota data from both the animal experiments, corrected for the variable distance (squared Bray Curtis) to their paired colon microbiota and the variable “study” (adjusted explained variation = 2.46%; *P* = 0.004). **(B)** Spearman correlation between eating scores of EF piglets and the relative abundance of *Escherichia-Shigella* in the rectal swab (experiment 1).

In a previous study, we have established that early feeding (of fibrous diet) in piglets accelerated the microbiome development over time (Choudhury et al., 2021a) and significantly altered the microbiota of the colon lumen (Choudhury et al., 2021b). The current study offered the possibility to evaluate the impact of early feeding on the mucosa-adhered population in rectal swabs, focussing especially on the genus *Escherichia-Shigella*, which includes pathogenic species that are strongly implicated in post-weaning diarrhoea (Gresse et al., 2017; Rhouma et al., 2017). The effect of early feeding could be detected in swab samples (Supplementary Figure 4A), with similar microbial groups associated with the EF group as reported earlier (Choudhury et al., 2021b). To further assess the impact of early feeding on mucosa-adhered population, we performed pRDA analysis corrected by the variables “Bray Curtis distance of the rectal swab to its paired colon sample” and “study,” using the data from both experiments. Intriguingly, the *Escherichia-Shigella* genus was found to be less abundant in EF compared to CON piglets (Figure 2A), suggesting that this post-weaning-diarrhoea-associated genus may be suppressed by early feeding. However, we also noted that this effect was almost exclusively driven by the first experiment (Supplementary Figures 4B,C). Nevertheless, the relatedness of the abundance of this genus with early feeding in the first experiment was further supported by the trending negative correlation (*r* = −0.52, *P* = 0.06) between actual eating behaviour of piglets [video scores from experiment 1; (Choudhury et al., 2021b)] and the relative abundance of *Escherichia-Shigella* (Figure 2B). These findings suggest that the early feeding intervention may affect this important group of bacteria, although this effect may be also dependent on additional and so far unknown environmental factors that differed between the two experiments we analysed here.

### Predicting the Degree of Representation of Mucosa-Adhered Microbiome (i.e., the Mucosity Factor) in Rectal Swab Samples

**FIGURE 3:**
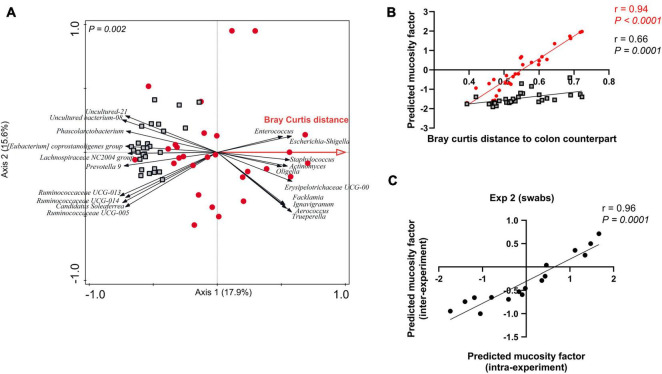
Predicting the mucosity factor in rectal swab samples. **(A)** Redundancy analysis (RDA) of the mucosity factor “Bray Curtis distance to colon counterpart” (adjusted explained variation = 14.8%; *P* = 0.002), in swab samples only (red dots) creating an ordination space that enables the prediction of the mucosity factor. Colon samples (black outlined squares) were added as supplementary in this figure. **(B)** Spearman correlation between “Bray Curtis distance to colon counterpart” and the position scores (or CaseR scores) of swab (red dots) and colon (black outlined square; added as supplementary) samples in the mucosity factor predicting ordination space. **(C)** Spearman correlation between the Predicted mucosity factor (CaseR scores) intra- vs. inter-experiment. For the inter-experiment CaseR scores, experiment 2 swab samples were added as supplementary samples in the prediction ordination space created by experiment 1 swab samples (Figure 3A).

As postulated above, the degree of mucosa-adhered microbiome presence in an individual swab sample appears to be reflected by its distance relative to the paired colon sample, which we refer to as the “mucosity factor.” We wanted to assess whether this mucosity factor determined for a subset of animals (for which both swab and colon samples are available) enables the prediction of the mucosity factor of rectal swabs that lack a paired colon sample. To this end, we performed RDA analysis using swab microbiota information to explain the variation with the Bray Curtis distance or “mucosity factor” (as explanatory variable). This analysis of Bray Curtis distance (Figure 3A) depicted very similar swab-associated microbes as found in Figure 1B, demonstrating that the right side of the ordination space includes samples which have a higher “mucosity factor” (or higher abundance of mucosa-adhered population), while the left side of the plot includes samples with lower mucosity factor (Figure 3A). The latter result is confirmed by adding the colon samples from the same experiment as supplementary samples (Figure 3A; black outlined squares) in the same ordination space (i.e., the colon samples do not influence the ordination space created), revealing that all colon samples were positioned at the left-side of the plot, illustrative of their low or even absent “mucosity factor” (Figures 3A,B). The positioning of a swab sample in the created ordination space is able to predict its mucosity factor, which was confirmed by the strong correlation (*r* = 0.94, *P* < 0.0001) of their positioning in this analysis (CaseR score in the RDA, which we refer to as “predicted mucosity factor”) and the measured Bray Curtis distance relative to the paired colon sample (i.e., the measured “mucosity factor”) (Figure 3B). The entire procedure employed on the data obtained from experiment 1 was repeated for experiment 2, generating very similar outcomes (Supplementary Figures 5A,B), thereby demonstrating the reliability of the predictive-ordination space created by swab microbiota data. Strikingly, the “predicted mucosity factor” was not only consistent within the same experiment, but also for swab samples collected from another experiment. For example, swabs from experiment 2 when placed as supplementary samples in the ordination space created using swab samples of experiment 1, have consistent “predicted mucosity factor,” which exemplifies the congruency of the mucosa adhered microbiota in these biologically independent but similarly designed animal experiments (Figure 3C and Supplementary Figures 3F, 5C). These findings demonstrate that we can predict the mucosity factor (or mucosa adhered microbiota) of individual swab samples, using a subset of paired swab-colon samples, which may even be applicable for swab samples obtained from a different experiment.

## Discussion

In a previous study (Choudhury et al., 2019), we evaluated rectal swabs as an alternative to faecal samples, to study the porcine microbiome development in early life. We concluded that regardless of the sample type (swab, faeces), the biological interpretation with respect to age-related microbiota was mostly comparable, although rectal swabs appeared to be enriched in mucosa-adhered microbial populations albeit to a variable degree. The variable degree of adhered populations in swab samples might be related to the length of time between the last defaecation and the swab sample collection. In the present study, we evaluated the exploitation of this variable degree of representation of the mucosa-adhered microbiome or “mucosity factor” in individual swab samples. In addition, we evaluated whether such approach could enable the prediction of the mucosa-adhered microbiome or mucosity factor in other swab samples.

The data presented illustrate that the swab microbiota tends to be more variable compared to the colon microbiota, which appeared to reflect the variable degree of representation of the mucosa-adhered microbial populations that are substantially less abundant in the colon microbiota. This conclusion is supported by the enrichment of the microbial genera such as *Escherichia-Shigella, Actinomyces, Trueperella, Peptostreptococcus, Anaerococcus, Peptoniphilus* which are known to inhabit the mucosal surfaces in the gastrointestinal tract. Strikingly, the most prominent microbial group associated with the mucosa-adhered microbiota (e.g., enriched in the swab compared to its paired colon sample) was the genus *Escherichia-Shigella*, which was virtually absent in colon content samples. This is in agreement with the facultative anaerobe character of the members of this genus, making them more likely colonisers of the mucosal tissues where elevated oxygen concentrations are present compared to the intestinal lumen (Gresse et al., 2017; Rhouma et al., 2017). Moreover, other porcine studies have also reported *Escherichia* to be less abundant in the colon content compared to the other intestinal/mucosal locations (Mann et al., 2014; Kelly et al., 2017; Gresse et al., 2019). Although 16S rRNA approaches are unable to distinguish different members of the *Escherichia-Shigella* genus, this microbial group includes a variety of opportunistic pathogens such as enterotoxigenic *Escherichia coli* (ETEC) that are considered the most common cause of porcine post-weaning diarrhoea (Rhouma et al., 2017; Gresse et al., 2019). These findings emphasise the importance of quantitatively assessing the mucosa-adhered populations in young piglets, to subsequently evaluate how these microbial populations could be influenced by (pre-weaning) dietary interventions (Kelly et al., 2017; Duarte and Kim, 2021; Klymiuk et al., 2021).

A comparative microbiota analysis of colon and rectal swab samples obtained from the same piglets, allowed us to assess the impact of a (fibrous-pre-weaning) diet intervention on the mucosa-adhered populations, including the abundance of the genus *Escherichia-Shigella*. The impact of diet on the mucosa-adhered microbiota composition was assessed by analysing the swab samples from EF and CON piglets using the Bray Curtis distance as a covariate in the RDA, in order to correct for the degree of representation of mucosa-adhered microbiome in the swabs. This approach revealed that in our first experiment the mucosa-adhered *Escherichia-Shigella* population was suppressed by early life feeding, which was supported by the notion that the amount of fibrous-feed consumption (pre-weaning) is correlated to the level of suppression of this genus. However, this effect of pre-weaning fibrous-feed consumption was not observed in a second study that followed a similar design, indicating that further studies are needed to establish whether this dietary intervention regiment is able to suppress this genus, and eventually reduce post-weaning diarrhoea. Nevertheless, the sampling and data-analysis approach we present here would strongly facilitate such follow-up studies to evaluate the role of early feeding diet or other interventions in suppressing the *Escherichia-Shigella* genus in the mucosa-adhered microbiota.

We also demonstrated that the ordination space created only by the swab samples, is able to predict the “mucosity factor” or the degree of representation of the mucosa-adhered microbiota in other samples. The predictive-ordination space could successfully classify the samples quantitatively based on their “mucosity factor.” The predictability not only holds for samples collected within the same experiment but also collected from the other experiment, which is in agreement with the congruency of the microbial genera that differentiate the swab samples from their colon content counterparts identified in both experiments. However, it is important to realise that both the experiments included in our analysis were similarly designed and were executed in the same animal facility. Thereby, we cannot estimate the impact of different housing facilities or different intervention designs on the mucosa-adhered microbiome composition. Nevertheless, and irrespective of the influence of these potential confounding factors (housing facilities and intervention design), the analysis strategy we present enables large piglet cohort studies (e.g., including intervention and control groups) for the evaluation of interventions on the mucosa-adhered microbiota. Such studies would only require the sampling of colon content [or faecal samples; (Choudhury et al., 2019)] as well as rectal swabs in a subset of piglets to allow for the comparative analysis of this subset (creation of the ordination space) in order to predict the mucosity factor in rectal-swab samples collected from all other piglets in the cohort.

## Conclusion

Rectal swab samples can be employed to assess the mucosa-adhered populations, while informing also about the luminal microbiota. The mucosity factor that reflects the degree of mucosa-adhered microbiota in individual swab samples can be deciphered using the Bray Curtis distance of paired rectal swab and colon content (or faecal) samples. Importantly, such paired information is only required for a subset of the piglets in the study cohort to predict the mucosity factor in all other rectal swabs collected, or in studies that are more similarly executed could even employ paired swab-faecal sample sets obtained from another study. Our approach opens avenues to investigate the (longitudinal) impact of dietary interventions on the composition of the mucosa-adhered microbiota, without the need to sacrifice animals. The relevance of this possibility is underpinned by prominent presence of the post-weaning diarrhoea causing *Escherichia-Shigella* genus in the mucosa-adhered microbiota. Moreover, the abundance of this genus within the mucosa-adhered microbiota could be influenced by diet, although further research is needed to unequivocally determine whether pre-weaning supplementation (like the fibrous-feed we employed in this study) are able to achieve the suppression of this problem causing genus.

## Data Availability Statement

The datasets presented in this study can be found in online repositories. The names of the repository/repositories and accession number(s) can be found below: https://www.ncbi.nlm.nih.gov/bioproject/PRJNA687128; https://www.ncbi.nlm.nih.gov/bioproject/PRJNA775018.

## Ethics Statement

The animal study was reviewed and approved by the Wageningen University & Research (AVD104002016515).

## Author Contributions

RC performed the experiments, processed samples, analyzed sequencing data, statistics, and prepared the figures. RC and MK interpreted the data and wrote the original draft of the manuscript. Both authors approved the final manuscript.

## Conflict of Interest

The authors declare that the research was conducted in the absence of any commercial or financial relationships that could be construed as a potential conflict of interest.

## Publisher’s Note

All claims expressed in this article are solely those of the authors and do not necessarily represent those of their affiliated organizations, or those of the publisher, the editors and the reviewers. Any product that may be evaluated in this article, or claim that may be made by its manufacturer, is not guaranteed or endorsed by the publisher.

## Footnotes

1
www.graphpad.com
