# Supplementary material for: Assessing the Impact of Diet on the Mucosa-Adhered Microbiome in Piglets Using Comparative Analysis of Rectal Swabs and Colon Content
Source: Front Microbiol. 2022 Feb 22;13:804986. doi: 10.3389/fmicb.2022.804986 (PMC8902596; doi:10.3389/fmicb.2022.804986)
Supplement: Supplementary file 1 [file Data_Sheet_1.PDF]

<u>Supplemental information belonging with:</u>

Assessing the impact of diet on the mucosa-adhered microbiome in piglets using comparative analysis of rectal swabs and colon content

R. Choudhury, M. Kleerebezem

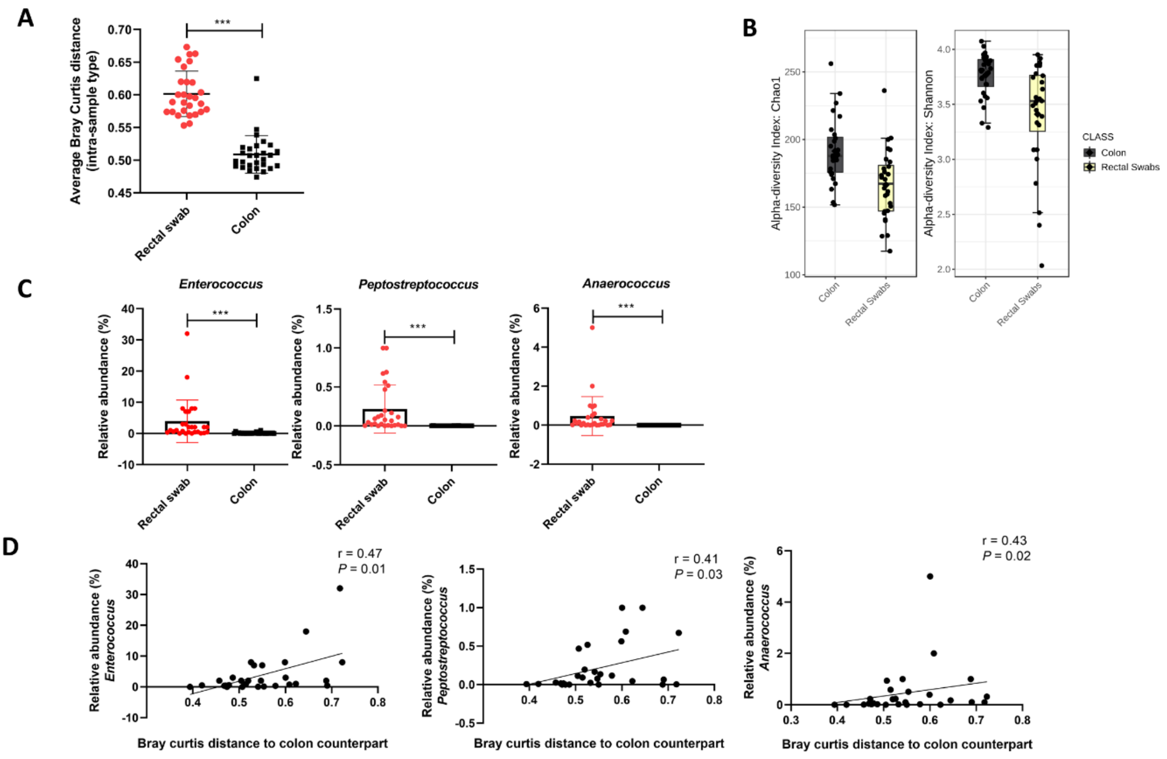

Supplementary figure 1: (**A**) Intra–sample type distance (Bray Curtis) in rectal swab and colon samples. (**B**) Comparing the alpha diversity between colon and rectal swab samples based on Chao1 and Shannon indices ($P < 0.0001$). (**C**) Bar plots displaying relative abundance of enriched microbes in the rectal swab samples compared to the colon samples (Mann Whitney t test; ***, $P < 0.0001$). (**D**) Spearman correlation of the relative abundance of enriched microbes in the rectal swab samples and the distance (Bray Curtis) of the rectal swab with respect to the paired colon sample (collected from the same animal).

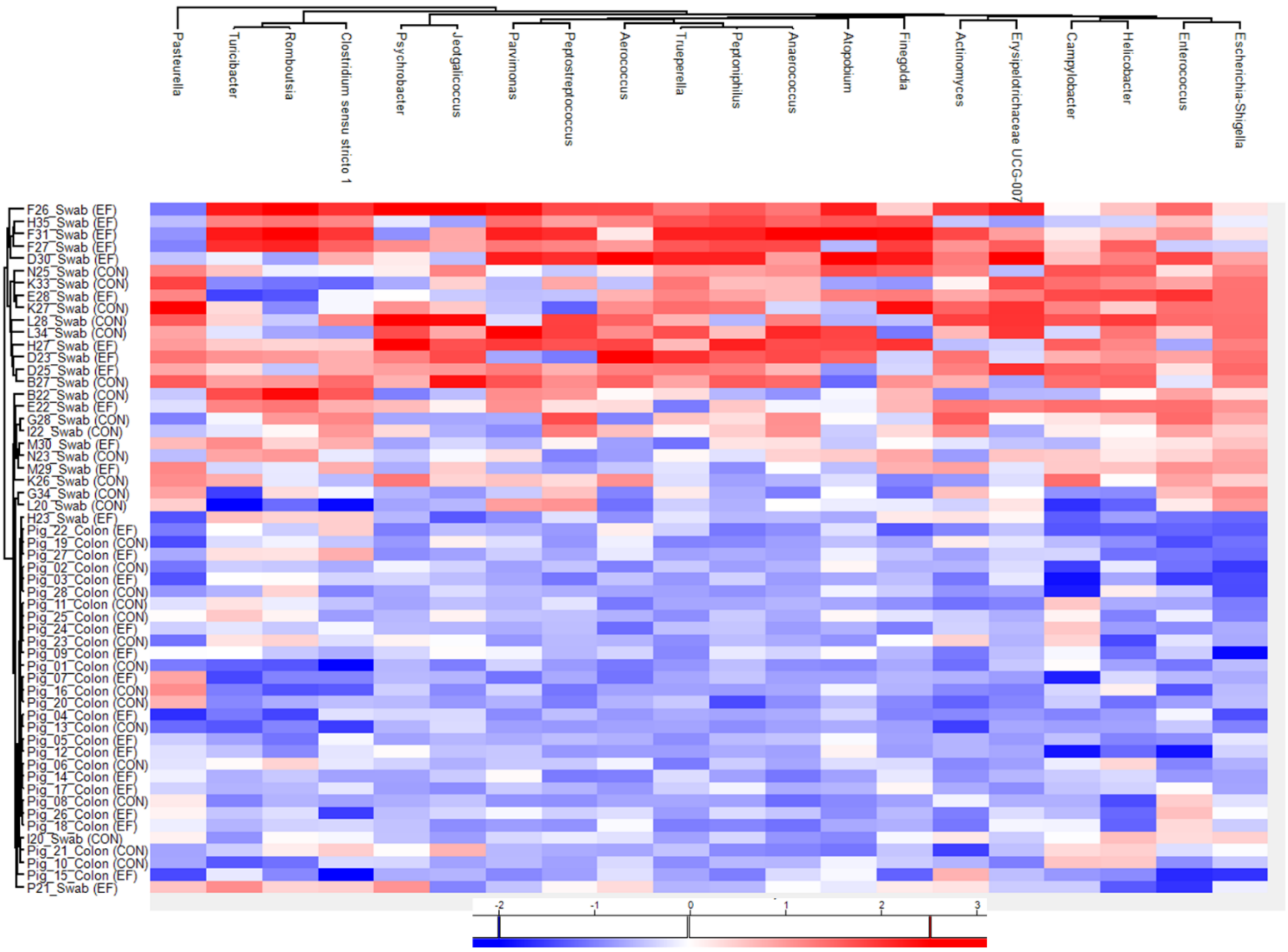

Supplementary figure 2: Heat map of the discriminative microbes (top 20 identified in redundancy analysis; **Figure 1B**) in rectal swabs and colon samples.

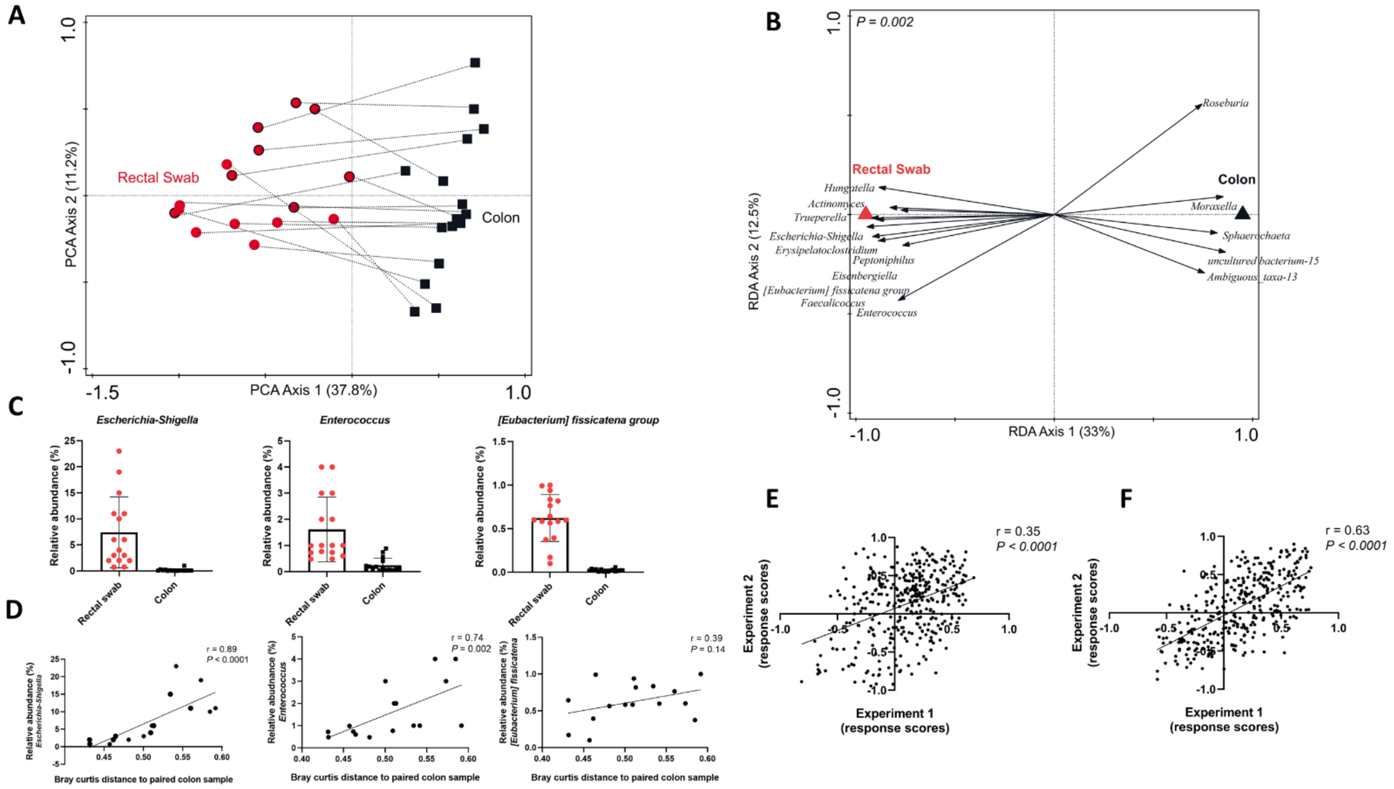

Supplementary figure 3: Reassessing the swab-colon microbiota in a replicate experiment 2. (**A**) Principal Component Analysis (PCA) of rectal swab [red circles; early fed piglets: red circles with black border) and colon (black squares) microbiota, with paired samples joined by dotted lines. (**B**) Redundancy analysis (RDA) of rectal swab and colon samples (P = 0.002), displaying discriminating microbial groups (response score > 0.60). (**C**) Bar plots of representative microbes enriched in the rectal swab samples compared to the colon samples (Mann Whitney t test; ***, P < 0.0001). (**D**) Spearman correlation between the relative abundance of representative microbes in the rectal swab sample and the (Bray Curtis) distance of the rectal swab with respect to the paired colon sample. (**E**) Spearman correlation between the microbial response scores of the two experiments (obtained from redundancy analysis of swab vs colon samples). (**F**) Spearman correlation between the microbial response scores of the two experiments (obtained from redundancy analysis of swab samples taking Bray Curtis distance as an explanatory variable).

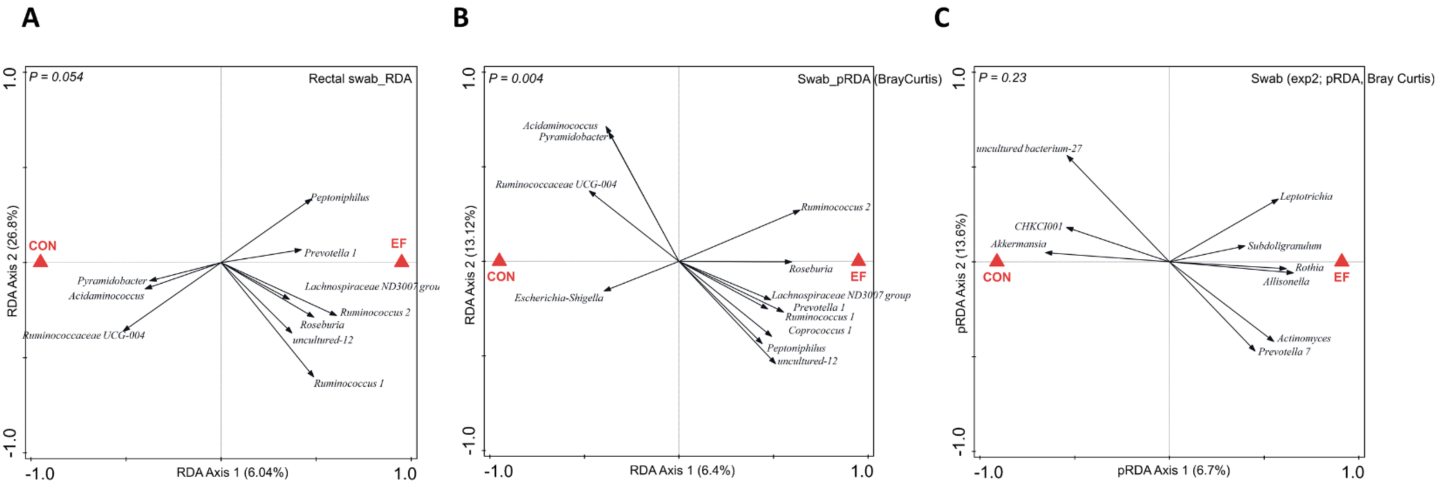

Supplementary figure 4: (A) Redundancy analysis of the fibrous diet intervention (early fed or EF vs control or CON) in piglets employing rectal swab samples only (adjusted explained variation = 2.43%; *P* = 0.054). Partial redundancy analysis of EF vs CON corrected for the distance (squared Bray Curtis) to their paired colon, employing the rectal swab microbiota data from (**B**) experiment 1 (adjusted explained variation = 4.1%; *P* = 0.004) and (**C**) experiment 2 (adjusted explained variation = 1.64%; *P* = 0.23).

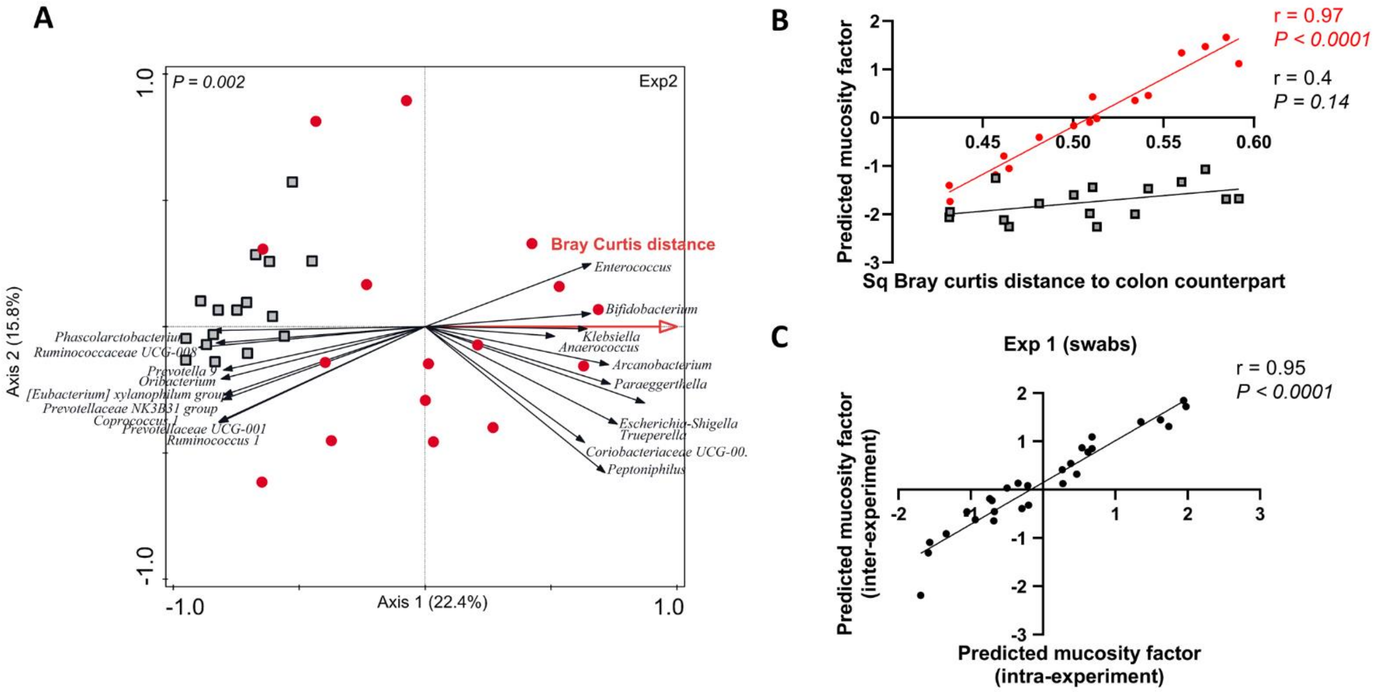

Supplementary figure 5: Reassessing the mucosity predictability in the replicate experiment 2. (A) Redundancy analysis of the mucosity factor “Bray Curtis distance to colon counterpart” in swab samples only (adjusted explained variation = 16.8%; *P* = 0.002), creating an ordination space for predicting mucosity factor. (**B**) Spearman correlation between “Bray Curtis distance to colon counterpart” and the position scores (or CaseR scores) of swab (red dots) and colon (black outlined square; added as supplementary) samples in the mucosity prediction ordination space. (**C**) Spearman correlation between the Predicted mucosity factor (CaseR scores) intra-experiment vs inter-experiment. For the inter-experiment CaseR scores, experiment 1 swab samples were added as supplementary in the prediction ordination space created by experiment 2 swab samples (opposite to what was done in Figure 3C).
